# Dietary Patterns and Renal Health Outcomes in the General Population: A Review Focusing on Prospective Studies

**DOI:** 10.3390/nu11081877

**Published:** 2019-08-13

**Authors:** Aparna S. Ajjarapu, Stefanie N. Hinkle, Mengying Li, Ellen C. Francis, Cuilin Zhang

**Affiliations:** 1 Division of Intramural Population Health Research, *Eunice Kennedy Shriver* National Institute of Child Health and Human Development, National Institutes of Health, Bethesda, MD 20817, USA; 2 National Institutes of Health Graduate Partnerships Program, Bethesda, MD 20817, USA; 3 Department of Public Health Sciences, Clemson University, Clemson, SC 29634, USA

**Keywords:** dietary pattern, diet, kidney, kidney disease, prevention

## Abstract

Healthy dietary patterns may promote kidney health and prevent adverse renal outcomes. Although reviews have summarized the findings from studies on dietary patterns for chronic kidney disease (CKD) management, less is known about dietary patterns for maintaining kidney health prior to CKD development. The current review summarized the results from observational studies from March 2009 to March 2019 investigating associations between dietary patterns and renal outcomes in the general population. The main renal outcome assessed was CKD (estimated glomerular filtration rate < 60 mL/min/1.73 m^2^). A total of twenty-six research articles met the inclusion criteria. Adherence to the Dietary Approaches to Stop Hypertension (DASH) and Mediterranean diets were significantly associated with a decreased risk of CKD in the majority of the studies. Furthermore, *a posteriori* “unhealthy” dietary patterns were associated with an increased risk of CKD. In conclusion, the findings from this review suggest that adherence to DASH and Mediterranean dietary patterns may be useful in promoting kidney health and preventing CKD in the general population. More studies, in particular among minorities, are warranted to investigate the role of diet, a potentially modifiable factor, in promoting kidney health.

## 1. Introduction

Chronic kidney disease (CKD), described as abnormalities in kidney structure and function [1], has a global prevalence of 13.4% [2]. CKD is associated with increased cardiovascular morbidity, premature mortality and decreased quality of life [3,4,5,6]. It is estimated that the United States spent 64 billion dollars in Medicare expenditures for CKD treatment in 2015 [7,8] and developed countries spend 2–3% of their annual health care budget on the treatment of end-stage renal disease (ESRD) [8,9]. Furthermore, the costs for treatment increase exponentially as CKD advances in stage [10]. Major risk factors for CKD include race, gender, age, diabetes, hypertension, and obesity [7]. Dietary factors are emerging risk factors for chronic diseases and exploring these factors could promote kidney health and prevent CKD, which is important for reducing the morbidities and the high health care costs associated with this disease.

The majority of previous studies on diet and renal function have focused on individual nutrients; however, nutrients are rarely consumed in isolation and meals consist of combinations of nutrients with complex interactions [11]. The study of dietary patterns, as opposed to individual nutrients, is more informative and relevant to public health and clinical practice as it accounts for nutrient interactions, cultural considerations, and eating habits and dietary pattern studies are more easily translatable into dietary guidelines [12]. Previous studies conducted among individuals with kidney disease have observed that components of healthy dietary patterns are associated with improved kidney function through decreasing cardiometabolic risk factors such as inflammation and endothelial dysfunction [13]. The low dietary acid load and important bioactive nutrients of healthy dietary patterns may be driving these associations and preventing renal impairment among these individuals [13,14].

Previous systematic reviews and meta-analyses based on studies among CKD patients investigating the role of dietary patterns for CKD management suggest that “healthy” diets such as the Mediterranean diet and a diet adhering to the Healthy Eating Index, are associated with improved renal health [15,16]. Several review articles have also focused on the effect of high and low protein diets for CKD management among CKD patients and healthy adults and suggest high protein diets may be detrimental to renal health [17,18,19,20,21]. Studies on dietary patterns for CKD prevention are emerging. Previous reviews have focused on studies conducted among individuals with kidney disease for the management and treatment of adverse renal outcomes. To the best of our knowledge, however, there is no up-to-date review on studies among the general population investigating dietary patterns and renal outcomes from a prevention perspective for CKD and other relevant kidney health outcomes. Furthermore, the findings from prior studies on this topic have been inconsistent, necessitating a review summarizing the literature. The current narrative review aims to systematically review emerging studies on the potential role of dietary patterns in the prevention of CKD, which may contribute to the development of dietary guidelines for promoting kidney health.

## 2. Materials and Methods

### 2.1. Eligibility Criteria

This review included studies published between March 2009 and March 2019 that investigated associations between dietary patterns and renal health outcomes among the general populations without major kidney diseases. Studies on *a priori*- or *a posteriori*-defined dietary patterns were included. The search was limited to the following renal health outcomes: CKD, albuminuria, proteinuria, glomerular hyperfiltration, estimated glomerular filtration rate (eGFR) decline, dialysis, death due to renal cause, kidney stones, urinary albumin-to-creatinine ratio (UACR), eGFR, and creatinine clearance rate. The primary outcome was CKD, defined as an eGFR < 60 mL/min/1.73 m^2^ [1]. Secondary outcomes included albuminuria and proteinuria, defined as a UACR 30–299 mg/g [22], dialysis, death due to renal causes, kidney stones, eGFR decline, which is a measure of the decrease in eGFR over a given period of time with rapid eGFR decline previously observed to be associated with renal impairment [23], glomerular hyperfiltration, which may precede CKD development and defined as an eGFR ≥ 120 mL/min/1.73 m^2^ [23], and creatinine clearance rate, increases in which have been associated with renal impairment [24].

Studies were excluded if they did not report primary data (review articles, editorials, non-research letters), were of a case-reports, case-series, or ecological design, were not conducted in humans, lacked dietary pattern data, renal function outcomes or measures of association (hazard ratios, odds ratios, relative risks, beta correlation coefficients). Studies were also excluded if they focused on a population with a disease, or if participants were younger than 21 years. Lastly, studies were excluded if they only investigated components of a dietary pattern, but not the whole dietary pattern in relation to renal outcomes.

### 2.2. Literature Search, Screening and Data Extraction

A pilot literature search was conducted; we observed that the majority of studies relevant to this review were published within the past 10 years. As such, PubMed and Embase were searched for relevant studies from March 2009 to March 2019. Search strategies were developed *a priori* for each database with assistance from a National Institutes of Health (NIH) librarian and the outline of the Preferred Reporting Items for Systematic Reviews and Meta-Analyses (PRISMA) 2009 flow diagram was followed to screen studies for this review. The search targeted titles and abstracts as well as subject headings. Search terms consisted of the renal outcomes, dietary patterns, and observational studies. We did not find any clinical trials in the literature search. The search strategy and search history are presented in Supplemental Table S1.

**Figure 1:**
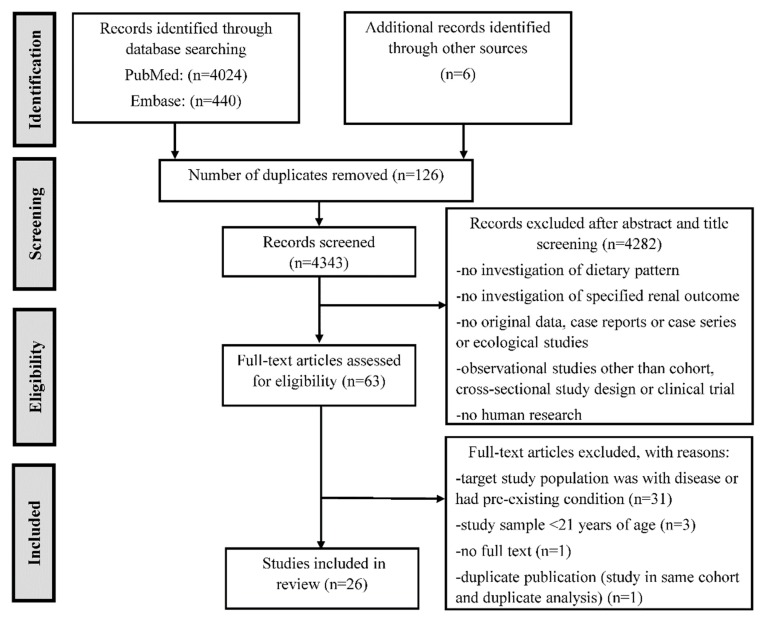
Flow diagram of study selection process.

After this literature search, the title and abstracts of the resulting records were screened for eligibility, and then the full text of the retained records were assessed for final inclusion decisions (Figure 1). Major studies that were published before 2009 and indexed in PubMed and the reference lists of relevant research articles that were not covered in the literature search were also searched for. Relevant data were abstracted from the retained publications using a standard form. The major findings from the included studies are summarized by different dietary patterns.

## 3. Results

A total of 4343 unique records were obtained from the literature search. After title and abstract screening, 61 eligible records remained. After the full text review, 20 articles were included. Six additional articles were also included; five were found from the bibliographies of the included full text articles and one was found from an informal PubMed search of major relevant studies published before 2009 (Figure 1). The main characteristics of the prospective (*n* = 16) and cross-sectional studies (*n* = 11) are presented in Table 1 and Supplemental Table S2, respectively. Descriptions of the *a priori* and *a posteriori* dietary patterns are presented in Supplemental Tables S3 and S4, respectively. Below, we describe the major findings from this review by different dietary patterns.

### 3.1. Dietary Approaches to Stop Hypertension (DASH) Diet

The DASH diet is characterized by a high intake of fruits, vegetables, nuts and legumes, low-fat dairy products, and whole grains and a low intake of sodium, sweetened beverages, and red and processed meats [41]. In the studies included in this review, the DASH diet score was calculated as the sum of 6, 8 or 9 individual component scores [42,43] (Supplemental Table S3).

Ten studies examined the DASH diet as the exposure. Among these studies, CKD incidence or prevalence was assessed as the outcome in five studies [30,34,35,44,45], incident kidney stones [32,33], microalbuminuria [25,31], or eGFR decline [25,35] were assessed in two studies and dialysis, death due to renal cause, and a composite outcome of dialysis and death due to renal cause were assessed in one study [26].

**Figure 2:**
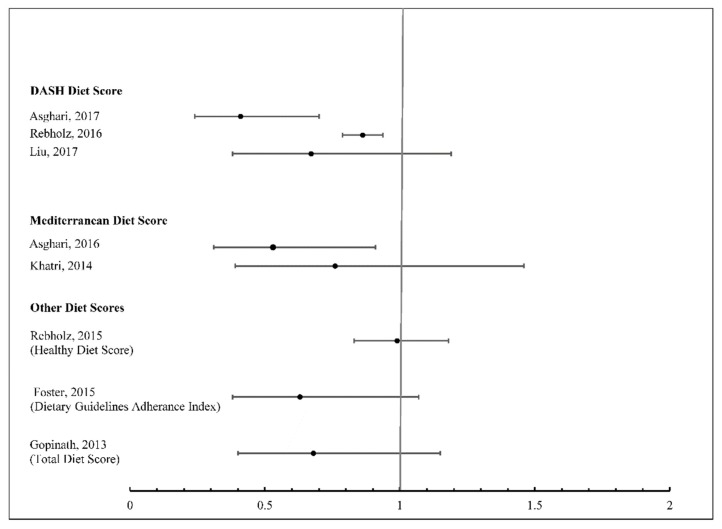
*A priori* dietary patterns and risk of chronic kidney disease in prospective studies ^1^. ^1^ The association measures compared the highest and lowest categories of diet scores with the lowest category of the diet score as the reference.

Three prospective studies investigated associations between the DASH diet and incident CKD [30,34,35] (Figure 2). Two of these studies, the Atherosclerosis Risk in Communities (ARIC) study and the National Institute on Aging, Healthy Aging in Neighborhoods of Diversity across the Life Span Study (NIA-HANDLS) were conducted in the United States, and one study, the Tehran Lipid and Glucose Study (TLGS), was conducted in Iran. The TLGS [34] (total N = 1630, mean age of 42.8 years) and the ARIC study [30] (total N = 14,882, age range of 45 to 65 years) observed significant decreases in the risk of incident CKD associated with greater DASH diet adherence.

Two cross-sectional studies investigated associations between the DASH diet and prevalent CKD [44,45], which were conducted in the United States and Korea. The Korean National Health and Nutrition Examination Survey [45] reported significant decreases in the risk of prevalent CKD when comparing the highest to the lowest level of DASH diet adherence. In the NIA-HANDLS (total N = 1534, mean age of 48 years), a lower risk of CKD associated with greater DASH diet adherence was also observed, but only among participants who were in poverty [35] and not overall. However, the inference of the findings from these studies was hindered by their cross-sectional design and ambiguous temporal relationship.

In addition to CKD, prospective studies were conducted on other renal health outcomes in association with the DASH diet. In the Nurses’ Health Study I (NHSI), the Nurses’ Health Study II (NHSII), and the Health Professionals Follow-up Study [32,33], a greater adherence to the DASH diet was associated with significant decreases in the risk of kidney stones. In the Coronary Artery Risk Development in Young Adults Study [31], greater adherence to the DASH diet was significantly associated with a decreased risk of microalbuminuria, yet this association was not observed in the NHSI [25]. Among the two studies examining DASH diet and eGFR decline, the NHSI reported a significant decreased risk of eGFR decline, [25] while the NIA-HANDLS reported no significant association [35]. The National Institutes of Health-American Association of Retired Persons Diet and Health (NIH-AARP) study observed significant decreases in the risk of death due to renal cause and a composite renal outcome of death due to renal cause and initiation of dialysis when comparing the lowest to the highest quintile of DASH diet adherence [26].

In summary, the majority of studies found that higher DASH scores were associated with lower risk of poor renal outcomes. The associations between higher DASH score and lower risk of CKD and kidney stones were generally consistent across studies, while the associations with eGFR and microalbuminuria were less consistent.

### 3.2. Mediterranean Diet

The Mediterranean diet is characterized by a high intake of non-refined cereals, fruits and nuts, vegetables, legumes, high ratio of monounsaturated fatty acids (MUFA) to polyunsaturated acids (PUFA), high to moderate intake of fish and low to moderate intake of red meat, poultry, full-fat dairy products, and alcohol [46,47]. In the studies included in this review, the Mediterranean diet was calculated as the sum of 8, 9 or 11 individual component scores [48,49,50] (Supplemental Table S3).

Five studies examined the Mediterranean diet as the exposure. Among these studies, CKD incidence was assessed as the outcome in two studies [27,28], and incident kidney stones [29], eGFR decline [28], the creatinine clearance rate [51], dialysis, death due to renal causes, and a composite outcome of dialysis and death due to renal causes [26] were assessed in one study.

Two prospective studies, the TLGS in Iran and the Northern Manhattan Study (NOMAS) in the United States, investigated associations between the Mediterranean diet and incident CKD [27,28] with follow-up durations of 6.1 and 6.9 years, respectively. Among these two studies, the TLGS reported a significant decreased risk of incident CKD [27], while the NOMAS reported no significant association when comparing the lowest to the highest category of Mediterranean diet adherence [28].

As for other renal health outcomes, in the SUN project, a prospective study in Spain, Mediterranean diet adherence was associated with a decreased risk of kidney stones [29]. The ATTICA study in Greece did not report any significant associations between the Mediterranean diet and the creatinine clearance rate [51]. The NIH-AARP study reported significant decreases in death due to renal causes and a composite outcome of death due to renal causes and initiation of dialysis when comparing the lowest to the highest categories of Mediterranean diet adherence [26]. No significant associations of the Mediterranean diet with eGFR decline [28] or the creatinine clearance rate [51] were reported.

In summary, a higher adherence to the Mediterranean diet was significantly associated with decreased risk of CKD, kidney stones, and death due to renal causes in some studies. However, studies in this regard are limited and more studies to replicate these findings are warranted.

### 3.3. Vegetarian Diet

Four studies examined the vegetarian diet as the exposure. Among these studies, CKD prevalence was assessed as the outcome in two studies [40,52], and kidney stones [39] and eGFR [53] were each assessed in one study. Descriptions of the vegetarian diets examined in these studies are presented in Supplemental Tables S3 and S4.

The TLGS, the only prospective study on CKD, reported significant decreases in risk of incident CKD when comparing the highest to the lowest tertial of the “lacto-vegetarian” diet [40]. A cross-sectional study in Taiwan also reported that “vegan” and “ovo-lacto vegetarian” diets were associated with significant decreases in risk of CKD compared to an “omnivore” diet [52]. As for other kidney health outcomes, the European Prospective Investigation into Cancer and Nutrition study was a prospective study that reported significant decreases in risk of incident kidney stones when comparing vegetarian and to a meat eaters diets [39].

In summary, studies on vegetarian diets and kidney health are sparse. Existing data appear to suggest that a vegetarian-type diet pattern was associated with a significantly decreased risk for CKD and kidney stones.

### 3.4. Other Diet Scores

Five studies examined other diet scores as the exposure. Among these studies, CKD incidence or prevalence was assessed as the outcome in three studies [37,38,54] and dialysis, death due to renal cause, and a composite outcome of dialysis and death due to renal cause [26], eGFR decline [37], and eGFR [55] were each assessed as an outcome in one study. Descriptions of the diet scores assessed in these studies are presented in Supplemental Table S3.

Three prospective studies investigated associations between the American Heart Association’s (AHA) Life’s Simple 7 Healthy Diet Score [36], the Dietary Guidelines Adherence Index (DGAI) [37], and the Total Diet score (TDS) [38] and CKD (Figure 2). In the Framingham Offspring Cohort study, greater adherence to the DGAI was associated with a significant decreased risk of CKD [37]. No significant associations for the AHA Healthy Diet Score, or the TDS, were reported [36,38].

In the NIH-AARP study, the Alternative Healthy Eating Index and Healthy Eating Index but not the Recommended Food Score were significantly associated with a decreased risk of death due to renal cause and a composite outcome of death due to renal cause and dialysis [26]. No associations between the DGAI and eGFR decline were observed in the Framingham Offspring Cohort Study [37]. In a cross-sectional analysis of the Uppsala Longitudinal Study of Adult Men and the Prospective Investigation of Vasculature in Uppsala Seniors in Sweden, the Adapted Dietary Inflammatory Index (ADII) was associated with a significantly lower eGFR [55]. However, inference from the findings from this study was hindered by the cross-sectional design.

In summary, although some diet scores, such as the DGAI, were significantly associated with a decreased risk of adverse renal outcomes, more studies on each diet score are needed to make any definitive conclusions.

### 3.5. A Posteriori Diet Patterns

Six studies examined *a posteriori* dietary patterns as the exposure. Among these studies, CKD was assessed as the outcome in four studies [40,56,57,58], microalbuminuria was assessed in two studies [25,59], and eGFR decline [25] and UACR [59] were each assessed as an outcome in one study. Descriptions of the *a posteriori* dietary patterns are presented in Supplemental Table S4.

The TLGS was the only prospective study and observed that a “High fat, high sugar” diet pattern was significantly associated with an increased risk of CKD [40]. Three cross-sectional studies examined CKD as an outcome. Among these studies, the National Health and Nutrition Examination Survey observed that a “Minerals and Vitamins” diet pattern was associated with a significant decrease in risk of CKD [56]. In the Multiethnic Study of Atherosclerosis (MESA), a “Whole grains and fruit” diet pattern was associated with a significantly decreased risk of microalbuminuria and was inversely related to UACR [59]. In the China Health and Nutrition Examination Survey, a “Traditional Southern” diet pattern was associated with a significantly increased risk of CKD, while a “Modern” diet pattern was associated with a significantly decreased risk of CKD [57]. In the Irish Nun and Eye Study, an “Unhealthy” diet pattern was associated with a significantly increased risk of CKD [58].

In NHSI, greater adherence to a “Western” diet pattern was associated with an increased risk for microalbuminuria but not eGFR decline.

In summary, “unhealthy” *a posteriori* dietary patterns were associated with significant increases in risk of CKD but the associations between “healthy” *a posteriori* dietary patterns and CKD risk were less consistent. More prospective studies are needed to confirm these findings.

## 4. Discussion

In general, studies on dietary patterns and CKD and related adverse renal outcomes in the general population are limited. The majority of studies were conducted on *a priori* dietary patterns, with the DASH diet being the most commonly investigated. The DASH diet appeared to be associated with improved kidney function although there was variability in the associations. Less evidence exists on the Mediterranean and vegetarian dietary patterns but the results from the few available prospective studies suggest a protective association with kidney function. A small number of studies have been conducted on other diet scores which generally found that healthier diets were associated with improved kidney health but more studies on each particular diet score are needed. Among the *a posteriori* dietary patterns, the “unhealthy” patterns were all significantly associated with adverse renal outcomes, whereas only a small number of the “healthy” dietary patterns were significantly associated with a reduced risk for kidney disease.

Although the precise underlying molecular mechanisms are unclear, the associations between healthful dietary patterns, such as the DASH and Mediterranean diet, and lower risk of adverse renal outcomes summarized in this review are biologically plausible. The DASH diet has been recommended by the American Heart Association for the management of hypertension and cardiovascular disease [41] and thus could improve kidney health by reducing cardiometabolic risk factors such as decreasing high blood pressure [60], oxidative stress [61], and endothelial dysfunction [62] and improving plasma lipid levels [63] and insulin sensitivity [64]. The omega-3 fatty acid content of the Mediterranean diet, which is rich in fish, nuts, and olive oil, could improve kidney health through the high monounsaturated fatty acid to saturated fatty acid ratio, which has been associated with improved plasma lipid profiles and insulin resistance and reduced blood pressure [63,65,66].

Biomarkers of inflammation have been linked to the progression of kidney disease [67]. Diets rich in whole grains, fruits, vegetables, and nuts, such as the DASH, Mediterranean, vegetarian and “Healthy” diet patterns, have been inversely associated with markers of inflammation including CRP [68] and soluble ICAM-1 [69], while “unhealthy” diet patterns, such as diets high in fats and processed foods, have been directly associated with elevated markers of inflammation including CRP [68], ICAM-1 [69], and VCAM-1 [70]. Fruits, vegetables, and nuts are also rich in important antioxidant compounds (vitamins, selenium, polyphenol, magnesium) that may improve endothelial function [71]. Furthermore, plant-derived foods (fruits, vegetables, legumes, whole grains) are high in fiber and have a low glycemic index and load which may reduce CKD risk factors such as obesity and diabetes [72,73].

Diets rich in plant protein, magnesium, and potassium and low in animal protein, such as the DASH, Mediterranean, vegetarian, and “Healthy” dietary patterns, have a low dietary acid load [74,75]. Animal experiments and human studies suggest that a lower dietary acid load is associated with a slower eGFR decline [74,75,76,77]. Mechanisms underlying the association of dietary acid load and kidney injury may include reducing pro-fibrotic factors such as angiotensin II, endothelin-1 and aldosterone thereby lowering the activation of the renin-angiotensin-aldosterone system [78,79,80] and decreasing the workload of the nephrons. Vegetable proteins may also contain beneficial antioxidants, such as isoflavones, which could reduce endothelial dysfunction [81].

On the other hand, unhealthy dietary patterns, such as the “western-style diet”, “High fat, high sugar”, “Traditional Southern”, and “Unhealthy” diet patterns, are characterized by high intake of meat, sugar desserts, high fat foods, high-sugar drinks, and refined grains, and may adversely affect renal function through impairment of renal vascularization, increased steatosis, impaired hormonal regulation, and increased inflammation [82].

It should be noted that the populations in the studies included in this review generally reported very low incidences of chronic diseases that are major risk factors for CKD such as diabetes, hypertension, and obesity. This suggests that at baseline, these populations were at low overall risk for adverse renal outcomes. Heterogeneity in data collection methods and analytical approaches across studies were noticed. The calculation of certain diet scores, such as the DASH and Mediterranean diet scores, differed between studies (Supplemental Tables S3 and S4). For example, in one cross-sectional study, certain components of the DASH score were adapted to the unique diet of the KNHANES study cohort in Korea [45]. Differences between studies in the assessment of renal outcomes were also observed. For example, different equations were used to determine the eGFR (i.e., Chronic Kidney Disease Epidemiology Collaboration vs. Modification in Diet and Renal Disease equations) that was used to stage CKD. In addition, some of the studies of small sample size did not have the power to adjust for a comprehensive list of potential confounders [44,51,53] and thus residual confounding in these studies is an important limitation that should be acknowledged. Furthermore, renal outcomes were not always clearly defined in the included studies. For example, the studies that assessed kidney stones as the outcome used self-report and the types of kidney stones assessed were frequently not clearly defined. In future studies, refined categorization of the clinical phenotype of the outcome may be important to clarify biological mechanisms driving the associations between dietary patterns and kidney stones. Also, the majority of available studies were conducted in white populations and only one study in this review investigated socioeconomic disparities in the associations between dietary patterns and renal outcomes, observing a higher risk among low-income individuals [44]. As such, more longitudinal and prospective cohort studies investigating dietary patterns and renal function among minority and disadvantaged populations are needed.

Our narrative review is the first to provide a summary of the findings from studies aimed at using dietary patterns for the prevention of adverse renal outcomes. Several findings from this review may be of interest to future studies wishing to investigate dietary patterns and renal function for CKD prevention. We observed that *a priori* healthy dietary patterns, such as the DASH diet and the Mediterranean diet, generally had significant protective associations towards renal function whereas the results on *a posteriori* dietary patterns were mixed. It should also be noted that “unhealthy” dietary patterns provide useful insight into which dietary factors should be avoided to maintain kidney health. Furthermore, this review revealed that studies calculating the DASH score were more frequent and less research exists on the Mediterranean diet, vegetarian diet, and other diet scores such as the AHEI, HEI, DGAI and TDS, and *a posteriori* dietary patterns. More prospective studies on these types of dietary patterns are needed.

## 5. Conclusions

In conclusion, the findings from this systematic review suggest that among the limited number of studies that have been conducted, following the DASH and Mediterranean diets may be useful in promoting kidney health and preventing CKD in the general population. More studies, in particular among minorities, are warranted to investigate the role of diet, a potentially modifiable factor, in kidney health.

## Figures and Tables

**Table 1:** Characteristics of the prospective studies of dietary patterns and renal outcomes.

First Author (Publication Year), Country	Population, Sample Size (Sex)	Age, Years	Outcome Ascertainment	Diet Assessment Method (No. of Items)	Follow-Up, Years	Outcome (Definition)	Dietary Pattern Identified (Method Used)	Association Measures with Outcomes (OR, RR, HR, sHR, IRR, and 95% CI) or Major Findings	Covariates in Fully Adjusted Model
Lin et al. (2011) [25], USA	NHS I, 3121 (women)	Median age of study sample: 67	-Urinary creatinine via modified Jaffe method using urine sample collected in 2000. -Urinary albumin via solid-phase fluorescence immunoassay using urine sample collected in 2000. -Plasma creatinine via modified kinetic Jaffe method from plasma samples collected in 1989 and 2000. -eGFR calculated via the MDRD equation.	FFQ (116). The dietary pattern was calculated from the cumulative average dietary pattern from FFQ on five visits from 1984–1998 for microalbuminuria and three visits from 1984–1990 for eGFR decline.	11	1. eGFR decline (≥30% between 1989 and 2000). 2. Microalbuminuria (UACR ≥ 25 mcg/mg).	(1) DASH-style diet (diet score) (2) Western diet (PCA) (3) Prudent (PCA)	**eGFR decline (OR)** Prudent Q1: 1.00 (ref.) Q2: 1.43 (1.04, 1.98) Q3: 1.07 (0.76, 1.51) Q4: 0.81 (0.55, 1.19) Western Q1: 1.00 (ref.) Q2: 1.22 (0.87, 1.73) Q3: 1.57 (1.08, 2.28) Q4: 1.48 (0.95, 2.30) DASH-Style Q1: 1.00 (ref.) Q2: 0.86 (0.63, 1.17) Q3: 0.79 (0.57, 1.09) Q4: 0.55 (0.38, 0.80) **Microalbuminuria (OR)** Prudent Q1: 1.00 (ref.) Q2: 0.89 (0.57, 1.42) Q3: 1.05 (0.66, 1.67) Q4: 0.97 (0.58, 1.61) Western Q1: 1.00 (ref.) Q2: 1.11 (0.68, 1.81) Q3: 1.12 (0.66, 1.92) Q4: 2.17 (1.18, 3.96) DASH-Style Q1: 1.00 (ref.) Q2: 0.80 (0.52, 1.23) Q3: 0.77 (0.49, 1.21) Q4: 0.71 (0.44, 1.14)	Age, BMI, hypertension, physical activity, energy intake, cigarette smoking, diabetes, cardiovascular disease, and ACE-inhibitor/ARB medication use
Smyth et al. (2016) [26], USA	NIH-AARP, 544,635 (both)	Mean age of study sample: 62.2 (SD: 5.4)	-Vital status ascertained from Social Security Administration Death Master File and NDI. -Self-reported dialysis was noted on the study follow-up questionnaire.	FFQ (124). Dietary pattern calculated from FFQ administered once at baseline from 1995–1996.	14.3	1. Composite death due to renal cause and initiation of dialysis (death where chronic renal disease was primary or contributing cause of death based on ICD coding system, censored 31st December 2011). 2. Self-reported dialysis questionnaire). 3. Death due to renal cause.	1. AHEI, 2010 (diet score) 2. HEI, 2010 (diet score) 3.MDS (diet score) 4. RFS (diet score) 5. DASH (diet score)	**Composite death due to renal cause and initiation of dialysis (sHR)** 1. AHEI-2010, HEI-2010, MDS, DASH but not RFS scores were significantly associated **Self-reported dialysis (sHR)** 1. None of the diet scores were significantly associated **Death due to a renal cause (sHR)** 1. AHEI-2010, HEI-2010, MDS, DASH but not RFS scores were significantly associated	Age, gender, BMI, smoking, education, ethnicity, physical activity, diabetes, heart disease, and stroke
Asghari et al. (2016) [27], Iran	TLGS, 1212 (both)	Mean age of study sample: 43.5 (SD: 9.4)	-Serum creatinine measured via Jaffe kinetic reaction with blood sample collected during fifth study visit between 2012–2015. -eGFR calculated via the MDRD equation.	FFQ (168). Dietary pattern calculated using FFQ administered during third study visit from 2006–2008.	6.1	1. Incident CKD (eGFR < 60 mL/min/1.73 m^2^).	MDS (diet score)	**Incident CKD (OR)** Q1: 1.0 (ref.) Q2: 0.95 (0.64, 1.40) Q3: 0.85 (0.55, 1.32) Q4: 0.53 (0.31, 0.91) P for trend: 0.030	Age, BMI, gender, smoking status, physical activity, total calorie intake, diabetes, hypertension, baseline eGFR
Khatri et al. (2014) [28], USA	NOMAS, 900 (both)	Mean baseline age of study sample: 64	-Serum creatinine measured via Jaffe reaction with blood sample collected between 2003–2008. -eGFR calculated via the MDRD equation.	FFQ (147). Dietary pattern calculated via FFQ administered at baseline in 1998.	6.9	1. Incident eGFR < 60 mL/min/1.73 m^2^ (started with eGFR ≥ 60 mL/min/1.73 m^2^ at baseline and had an eGFR < 60 mL/min/1.73 m^2^ at follow-up exam). 2. Upper quartile of eGFR decline. 3. Annualized eGFR decline.	MDS (diet score)	**Incident eGFR < 60 mL/min/1.73 m^2^ (OR)** Q1: 1.0 (ref.) Q2: 1.61 (0.88, 2.97) Q3: 0.51 (0.26, 1.02) Q4: 0.76 (0.39, 1.46) **Upper quartile of eGFR decline (OR)** Q1: 1.0 (ref.) Q2: 1.01 (0.62, 1.66) Q3: 0.49 (0.29, 0.82) Q4: 0.67 (0.41, 1.10) **Annualized change in eGFR (parameter estimate)** Q1: 1.0 (ref.) Q2: −0.37 (−0.86, 0.12) Q3: 0.11 (−0.37, 0.60) Q4: 0.20 (−0.28, 0.67)	Age, gender, BMI race/ethnicity, education, insurance status, physical activity, diabetes, smoking status, hypertension, LDL, HDL, baseline eGFR, and ACE inhibitor/ARB usage
Leone et al. (2017) [29], Spain	SUN Project, 16,094 (both)	Median baseline age of study sample: 36 (IQR: 28–46)	-Nephrolithiasis self-reported via study follow-up questionnaire (enough participants with at least one follow-up questionnaire by 2013).	FFQ (136). Dietary pattern calculated using FFQ administered at baseline in 1999.	9.6	1. Incident nephrolithiasis (was free of nephrolithiasis at baseline and reported nephrolithiasis diagnosis at study follow-up).	MDS (diet score)	**Incident nephrolithiasis (kidney stones) (HR)** T1: 1.0 (ref.) T2: 0.93 (0.79, 1.09) T3: 0.64 (0.48, 0.87) P for trend: 0.01	Gender, BMI, hypertension, diabetes, marital status, education, number of working hours per week, smoking, physical activity, time spent watching television, total energy intake, total water intake, calcium supplementation, vitamin D supplementation, and following a nutritional therapy
Rebholz et al. (2016) [30], USA	ARIC, 14,882 (both)	Age range of cohort: 45–65	-Serum creatinine measured via modified Jaffe Kinetic reaction using blood collected at any of the five follow-up exams.-eGFR calculated via the CKD-EPI equation. -Kidney disease related hospitalization or death based on (ICD)-9/10 codes identified via surveillance and linkage to the NDI. -End-stage renal disease (dialysis or transplantation) identified by linkage to the USRDS registry between baseline and follow-up exam.	FFQ (66). Dietary pattern calculated from cumulative average of diet from FFQ administered at baseline between 1987–1989 and third visit from 1993–1995.	23	1. Kidney disease (meeting one of the following: 1. < 60 mL/min/1.73 m^2^ with 25% eGFR decline at any follow-up study visit relative to baseline. 2. Kidney disease related hospitalization or death. 3. End-stage renal disease (dialysis or transplantation) at follow-up study relative to baseline.	DASH (diet score)	**Kidney disease (HR)** T1: 1.16 (1.07, 1.27) T2: 1.09 (1.00, 1.18) T3: 1.00 (ref.) P for trend: <0.001	Age, gender, race-center, education level, smoking status, physical activity, total caloric intake, baseline eGFR, overweight/obese status, diabetes, hypertension, systolic blood pressure, use of angiotensin-converting enzyme inhibitors or angiotensin receptor blockers
Chang et al. (2013) [31], USA	CARDIA, 2354, (both)	Age range of study sample: 28–40	-Urine albumin measured via nephelometric procedure with specific anti-albumin antibody using urine samples collected from 2000–2001, 2005–2006 or 2010–2011. -Urine and serum creatinine measured via Jaffe method using urine samples collected from 2000–2001, 2005–2006 or 2010–2011.	Interview-administered diet history. Dietary pattern calculated via diet info. calculated during study baseline year from 1995–1996.	15	1. Incident Microalbuminuria.	DASH (diet score)	**Incident Microalbuminuria (OR)** Q1 (lowest score): 2.0 (1.1,3.4) Q5: 0.0 (ref.)	Age, gender, baseline obesity, race, family history of kidney disease, education, total energy intake, and baseline (year-10) ACR
Taylor et al., (2009) [32], USA	NHS I, 94,108 (women)	Age range of cohort: 30–55	-Kidney stone self-reported via study questionnaire and diagnosis was confirmed through review of medical records.	FFQ (>130). Dietary pattern calculated using FFQ administered at baseline in 1986. FFQ info updated every 4 years.	18	1. Incident kidney stone accompanied by pain or hematuria. Follow-up years calculated from to baseline to the date of a kidney stone or death or to 31 May 2004.	DASH (diet score)	**Incident kidney stone accompanied by pain or hematuria (RR)** Q1: 1.00 (ref.) Q2: 0.89 (0.77, 1.02) Q3:0.76 (0.65, 0.87) Q4: 0.64 (0.54, 0.74) Q5: 0.58 (0.49, 0.68) P for trend: <0.001	Age, BMI, total energy intake, use of thiazide diuretics, fluid intake, caffeine, alcohol use, history of hypertension, and history of diabetes
Taylor et al. (2009) [32], USA	NHS II, 101,837 (women)	Age range of cohort: 25–42	-Kidney stone self-reported via study questionnaire and diagnosis was confirmed through review of medical records.	FFQ (>130) Dietary pattern calculated using FFQ administered at baseline in 1991. FFQ info updated every 4 years.	14	1. Incident kidney stone accompanied by pain or hematuria. Follow-up years calculated from baseline to the date of a kidney stone or death or to 31 May 2005.	DASH (diet score)	**Incident kidney stone accompanied by pain or hematuria (RR)** Q1: 1.00 (ref.) Q2: 0.92 (0.81, 1.03) Q3: 0.77 (0.68, 0.88) Q4: 0.75 (0.66, 0.86) Q5: 0.60(0.52, 0.70) P for trend: <0.001	Age, BMI, total energy intake, use of thiazide diuretics, fluid intake, caffeine, alcohol use, history of hypertension, and history of diabetes
Taylor et al. (2009) [32], USA	HPFS, 45,821 (men)	Age range of cohort: 40–75	-Kidney stone self-reported via study questionnaire and diagnosis was confirmed through review of medical records.	FFQ (>130) Dietary pattern calculated using FFQ administered at baseline in 1986. FFQ info updated every 4 years.	18	1. Incident kidney stone accompanied by pain or hematuria. Follow-up years calculated from baseline to the date of a kidney stone or death or to 31 January 2004.	DASH (diet score)	**Incident kidney stone accompanied by pain or hematuria (RR)** Q1:1.00 (ref.) Q2:0.89 (0.77, 1.01) Q3:0.77 (0.67, 0.89) Q4:0.64 (0.54, 0.74) Q5:0.55 (0.46, 0.65) P for trend: <0.001	Age, BMI, total energy intake, use of thiazide diuretics, fluid intake, caffeine, alcohol use, history of hypertension, and history of diabetes
Ferraro et al. (2017) [33], USA	NHS I, 59,740, (both)	Mean age of study sample: (52.9, SD: 7.1)	-Kidney stone self-reported via supplementary questionnaire and diagnosis was confirmed through review of medical records.	FFQ (>130). Dietary pattern calculated using baseline FFQ in 1986.	12.1	1. Incident kidney stone accompanied by pain or hematuria. Follow-up years of calculated from day of return of baseline questionnaire to incident kidney stone.	DASH-style diet (diet score)	**Incident kidney stone accompanied by pain or hematuria (IRR)** Q5:1.00 (ref.) Q4:0.98 (0.82, 1.17) Q3:1.22 (1.03, 1.44) Q2:1.32 (1.12, 1.56) Q1:1.47(1.25, 1.73)	Age, BMI, fluid, race, geographic area, use of thiazide diuretics, history of diabetes, and history of hypertension
Ferraro et al. (2017) [33], USA	NHS II, 90,449, (both)	Mean age of study sample: 36.6, (SD: 4.6)	-Kidney stone self-reported via supplementary questionnaire and diagnosis was confirmed through review of medical records.	FFQ (>130) Dietary pattern calculated using baseline FFQ in 1991.	11.3	1. Incident kidney stone accompanied by pain or hematuria. Follow-up years of calculated from day of return of baseline questionnaire to incident kidney stone.	DASH-style diet (diet score)	**Incident kidney stone accompanied by pain or hematuria (IRR)** Q5:1.00 (ref.) Q4:1.18 (1.04, 1.34) Q3:1.09 (0.96, 1.24) Q2:1.33 (1.18, 1.51) Q1:1.37 (1.21, 1.55)	Age, BMI, fluid, race, geographic area, use of thiazide diuretics, history of diabetes, and history of hypertension
Ferraro et al. (2017) [33], USA	HPFS, 41,937 (men)	Mean age of study sample: 54.2 (SD: 9.7)	-Kidney stone self-reported via supplementary questionnaire and diagnosis was confirmed through review of medical records.	FFQ (>130) Dietary pattern calculated using baseline FFQ in 1986.	11.5	1. Incident kidney stone accompanied by pain or hematuria. Follow-up years of calculated from day of return of baseline questionnaire to incident kidney stone.	DASH-style diet (diet score)	**Incident kidney stone accompanied by pain or hematuria (IRR)** Q5:1.00 (ref.) Q4:1.06 (0.90, 1.25) Q3:1.21 (1.04, 1.42) Q2:1.36 (1.17, 1.59) Q1:1.53 (1.31, 1.78)	Age, BMI, fluid, race, geographic area, use of thiazide diuretics, history of diabetes, and history of hypertension
Asghari et al. (2017) [34], Iran	TGLS, 1630 (both)	Mean age of study sample: 42.8 (SD: 11.2)	-Serum creatinine measured via Jaffe kinetic reaction method with blood sample collected during fifth study visit from 2012–2015. -eGFR calculated via the MDRD equation.	FFQ (168) Dietary pattern calculated using FFQ administered during third study visit from 2006–2008.	6.1	1. Incident CKD (eGFR < 60 mL/min/1.73 m^2^).	DASH-style diet (diet score)	**Incident CKD (OR)** Q1:1.00 (ref.) Q5:0.41 (0.24, 0.70) P for trend: <0.001	Age, gender, BMI smoking, total energy intake, eGFR, triglycerides, physical activity, hypertension, and diabetes
Liu et al. (2017) [35], USA	NIA-HANDLS, (1534) (both)	Mean age of study sample: 48	-Blood creatinine measured via modified kinetic Jaffe method and isotope dilution mass spectrometry using blood samples collected from 2009–2013.	24-hr food recall. Dietary pattern calculated via diet information collected at baseline from 2004–2008.	5	1. Incident CKD (follow-up eGFR < 60 mL/min/1.73 m^2^). 2. eGFR decline (>25% from baseline).	DASH (diet score)	**Incident CKD (RR)** High DASH accordance:1.00 (ref.) Low DASH accordance:1.49 (0.84, 2.63) **eGFR decline >25% (RR)** High DASH accordance:1.00 (ref.) Low DASH accordance: 1.36 (0.78, 2.37)	Age, gender, and race
Rebholz et al. (2015) [36], USA	ARIC, 14,832 (both)	Mean age of study sample: 54	-Blood creatinine measured via modified kinetic Jaffe method using blood samples on five study visits from 1990–1992, 1993–1995, 1996–1998, and 2011–2013. -eGFR calculated via the CKD-EPI equation.	FFQ (66) Dietary pattern calculated using baseline FFQ from 1987–1989.	22	1. Incident CKD (meets one of the following criteria 1. development of eGFR < 60 mL/min/1.73 m^2^ accompanied by ≥25% eGFR decline from baseline. 2. ICD 9/10 code for hospitalization due to CKD identified by surveillance of hospitalization and annual follow-up phone calls with study participants. 3. ICD 9/10 code for death due to CKD identified by linkage to the NDI. 4. ESRD identified by linkage to the US renal data system registry).	American Heart Association Life’s Simple 7 Healthy Diet Score (diet Score)	**Incident CKD (HR)** Poor healthy diet score: 1.00 (ref.) Intermediate healthy diet score: 1.02 (0.93, 1.13) Ideal healthy diet score: 0.99 (0.83, 1.18) P for trend: 0.55	Age, gender, race, and baseline eGFR
Foster et al. (2015) [37], USA	The Framingham Offspring Cohort, 1802, (both)	Mean age of study sample: 59	-Serum creatinine measured via modified Jaffe method using blood samples collected at baseline (1998–2001) and follow-up (2005–2008) and study visits. -eGFR calculated via the CKD-EPI equation.	FFQ (131). Dietary pattern calculated using FFQ administered at baseline from 1998–2001.	6.6	1. Incident eGFR < 60 mL/min/1.73 m^2^ (presence of eGFR <60 mL/min/1.73 m^2^ at follow up among participants with eGFR > 60 mL/min/1.73 m^2^ at baseline. 2. Rapid eGFR decline (annual decrease in eGFR ≥ 3 mL/min/1.73 m^2^).	DGAI (diet score)	**Incident eGFR < 60 mL/min/1.73 m^2^ (OR)** Q1 (lowest quality): 1.0 (ref.) Q2: 0.77 (0.47, 1.27) Q3: 0.52 (0.31, 0.89) Q4 (highest quality): 0.63 (0.38, 1.07) P for trend: 0.045 **Rapid eGFR decline (OR)** Q1 (lowest quality): 1.0 (ref.) Q2: 0.83 (0.56, 1.22) Q3: 0.73 (0.49, 1.10) Q4 (highest quality): 0.69 (0.45, 1.05) P for trend: 0.07	Age, gender, baseline eGFR, BMI, hypertension, diabetes, and dipstick proteinuria
Gopinath et al. (2013) [38], Australia	Blue Mountain Eye Study, 1952, (both)	Study sample age: ≥50	-Serum creatinine measured via isotope dilution mass spectrometry using blood samples collected at follow-up examination from 2002–2004 -eGFR calculated via the MDRD equation.	FFQ (145). Diet score calculated via FFQ administered at baseline from 1992–1994.	10	1.Incident CKD (eGFR < 60 mL/min/1.73 m^2^).	TDS (diet Score)	**Incident CKD (OR):** Q1: 1.00 (ref.) Q2: 0.99 (0.60, 1.64) Q3: 0.78 (0.46, 1.31) Q4: 0.68 (0.40, 1.15) P for trend: 0.10	Age, serum total cholesterol, hypertension, and history of diagnosed diabetes
Turney et al. (2014) [39], United Kingdom	EPIC, 51,336 (both)	Study sample age: ≥20	-Incidence of kidney stones determined by reviewing hospital records of study participants with (ICD)-9/10 codes.	FFQ (130). Dietary pattern from FFQ administered at baseline from 1993–1999.	716,105 person-years	1. Incidence of kidney stones. Follow-up calculated from the date of recruitment to the study to the earliest of date of kidney stone diagnosis, death or emigration.	1. Vegetarian diet	**Incident kidney stones (HR)** Meat eater (>100g/day): 1.00 (ref.) Vegetarian: 0.69 (0.48, 0.98) P for trend: 0.04	Smoking, BMI alcohol consumption, self-reported prior diabetes, and energy intake
Asghari et al. (2018) [40], Iran	TLGS, 1630 (both)	Mean age of study sample: 42.8 (SD: 11.2)	-Serum creatinine measured via Jaffe Kinetic reaction method with blood sample collected during fifth study visit from 2012–2015.	FFQ (168). Dietary pattern calculated using FFQ administered during third visit from 2006–2008.	6.1	1. Incident CKD (eGFR < 60 mL/min/1.73 m^2^).	1. Lacto-vegatarian (PCA) 2. Traditional Iranian (PCA) 3. High fat, high sugar (PCA)	**Incident CKD (OR)** Lacto-vegetarian T1: 1.0 (ref.) T2: 0.85 (0.62,1.15) T3: 0.57 (0.41, 0.80) P for trend: 0.002 Traditional Iranian: T1: 1.0 (ref.) T2: 1.26 (0.93, 1.72) T3: 0.91 (0.64, 1.32) P for trend: 0.698 High fat, high sugar: T1: 1.0 (ref) T2: 1.21 (0.87, 1.70) T3: 1.46 (1.03, 2.09) P for trend: 0.036	Age, gender, BI smoking total energy intake, physical activity, diabetes, and hypertension

Abbreviation of research studies: HR = Hazard ratio; sHR = sub-hazard ratio; RR = Relative risk ratio; OR = Odd’s ratio; IRR = Incident Rate Ratio; SD = standard deviation; FFQ = Food frequency questionnaire; NHS I = Nurses’ Health Study I; MDRD = The Modification of Diet in Renal Disease; UACR = urinary albumin-to-creatinine ratio; eGFR = estimated glomerular filtration rate; BMI = body mass index; DASH = Dietary Approach to Stop Hypertension; PCA = Principle component factor analysis; T = Tertial; Q = Quartile or quintile based on designation; ICD = international classification of diseases; AHEI = alternate healthy eating index; HEI = Healthy eating index; MDS = Mediterranean diet score; RFS = Recommended Food Score; DGAI = Dietary Guidelines Adherence Index; CKD-EPI = Chronic Kidney Disease Epidemiology Collaboration; TLGS = Tehran Lipid and Glucose Study; CKD = Chronic kidney disease; NOMAS = Northern Manhattan Study; SUN = Seguimiento Universidad de Navarra/University of Navarra Follow-up; ARIC = Atherosclerosis Risk in Communities Study; NDI = National Death Index; ICD = International Classification of Disease; CARDIA = Coronary Artery Risk Development in Young Adults; Nurses’ Health Study II; NIA-HANDLS = National Institute on Aging, Healthy Aging in Neighborhoods of Diversity across the Life Span; ESRD = End Stage Renal Disease; TDS = Total Diet Score; EPIC = European Prospective Investigation into Cancer and Nutrition; NIH-AARP = National Institutes of Health-American Association of Retired Persons Diet and Health.

## Supplementary Materials

The following are available online at https://www.mdpi.com/2072-6643/11/8/1877/s1, Table S1: Search strategy and history in PubMed and Embase; Table S2: Characteristics of cross-sectional studies of dietary patterns and renal outcomes; Table S3: Description of *a priori* dietary patterns; Table S4: Description of *a posteriori* dietary patterns.
