# Supplementary material for: Dietary Patterns and Renal Health Outcomes in the General Population: A Review Focusing on Prospective Studies
Source: Nutrients. 2019 Aug 13;11(8):1877. doi: 10.3390/nu11081877 (PMC6723758; doi:10.3390/nu11081877)
Supplement: Supplementary file 1 [file nutrients-11-01877-s001.pdf]

Online Supporting Information

Dietary patterns and renal health outcomes in the general population: a review focusing on prospective studies

Aparna S. Ajjarapu, BA; Stefanie N. Hinkle, PhD; Mengying Li, PhD; Ellen C. Francis, MS; Cuilin Zhang, MD, PhD, MPH

Ajjarapu et al. Dietary patterns and renal health outcomes in the general population: a review focusing on prospective studies. Online Supporting Information.

Supplemental Table 1. Search strategy and history in PubMed and Embase

#	Query	Items
**PubMed Search**		
#1	Search (("Guideline Adherence"[Mesh] AND (diet OR food OR eating OR eat OR dietary OR feeding OR nutrition OR nutrient*)) OR (adherence AND (nutrient* OR nutrition OR diet OR dietary OR food OR eat OR eating) AND (guideline* OR guidance OR recommendation*)) OR (dietary score* OR adequacy index* OR kidmed OR Diet Quality Index* OR Food Score* OR Diet Score* OR MedDietScore OR Dietary Pattern Score* OR "healthy eating index") OR ((index*[ti] OR score*[ti] OR indexes OR scoring[ti] AND indices[ti]) AND (dietary[ti] OR nutrient*[ti] OR eating[tiab] OR food[ti] OR food[mh] OR diet[ti] OR diet[mh]) AND (pattern* OR habit* OR profile*))) OR (("diet quality" OR dietary pattern* OR diet pattern* OR eating pattern* OR food pattern* OR eating habit* OR dietary habit* OR food habit* OR dietary profile* OR food profile* OR diet profile* OR eating profile* OR dietary guideline* OR dietary recommendation* OR food intake pattern* OR dietary intake pattern* OR diet pattern* OR eating style*) OR (DASH OR (dietary approaches to stop hypertension) OR "Diet, Mediterranean"[Mesh] OR vegan* OR vegetarian* OR "Diet, Vegetarian"[Mesh] OR "prudent diet" OR "western diet" OR omniheart OR (Optimal Macronutrient Intake Trial to Prevent Heart Disease) OR ((Okinawa* OR "Ethnic Groups"[Mesh] OR "plant based" OR Mediterranean[tiab]) AND (diet[mh] OR diet[tiab] OR food[mh]))))) OR diet[mh] OR (diet[tiab] AND (Paleolithic[tiab] OR vegan[tiab] OR macrobiotic[tiab] OR high-fat[tiab] OR high-protein[tiab] OR low-carbohydrate[tiab] OR fat-restricted[tiab] OR low-fat[tiab] OR ketogenic[tiab] OR atherogenic[tiab] OR fads[tiab] OR fad[tiab] OR "caloric restriction"[tiab]) OR food[mh] OR fast foods[mh] OR "fast food"[tiab] OR diet[mh] OR diet*[tiab] OR edible grain[mh] OR "whole grain"[tiab] OR "whole grains"[tiab] OR fruit[mh] OR vegetables[mh] OR fruit*[tiab] OR vegetable*[tiab] OR legume*[tiab] OR fish[tiab] OR poultry[tiab] OR "dietary pattern"[tiab] OR "dietary patterns"[tiab] OR functional food[mh] OR soy[tiab] OR soy foods[mh] OR nuts[tiab] OR meat[mh] OR dairy products[mh] OR eggs[mh] OR diet, diabetic[mh]	1203230
#2	Search albuminuria[mh] OR albuminuria[tiab] OR microalbuminuria[tiab] OR glomerular filtration rate[mh] OR glomerular filtration rate*[tiab] OR GFR[tiab] OR kidney function[tiab] OR "kidney dysfunction"[tiab] OR kidney/physiopathology[mh] OR kidney[ti] OR renal[ti] OR	582987

	proteinuria[tiab] OR urinary albumin-creatinine[tiab] OR egfr[tiab] OR "kidney disease"[ti] OR "kidney diseases"[ti] OR renal insufficiency, chronic[mh] OR "glomerular filtration rate"[MeSH Terms] OR proteinuria[mh] OR albuminuria[mh] OR renal insufficiency[mh] OR renal outcome*[tiab]	
#3	Search "Clinical Trial" [PT:NoExp] OR "clinical trial, phase i"[pt] OR "clinical trial, phase ii"[pt] OR "clinical trial, phase iii"[pt] OR "clinical trial, phase iv"[pt] OR "controlled clinical trial"[pt] OR "multicenter study"[pt] OR "randomized controlled trial"[pt] OR "Clinical Trials as Topic"[mesh:noexp] OR "clinical trials, phase i as topic"[MeSH Terms:noexp] OR "clinical trials, phase ii as topic"[MeSH Terms:noexp] OR "clinical trials, phase iii as topic"[MeSH Terms:noexp] OR "clinical trials, phase iv as topic"[MeSH Terms:noexp] OR "controlled clinical trials as topic"[MeSH Terms:noexp] OR "randomized controlled trials as topic"[MeSH Terms:noexp] OR "early termination of clinical trials"[MeSH Terms:noexp] OR "multicenter studies as topic"[MeSH Terms:noexp] OR “Double-Blind Method”[Mesh] OR ((randomised[TIAB] OR randomized[TIAB]) AND (trial[TIAB] OR trials[tiab])) OR ((single[TIAB] OR double[TIAB] OR doubled[TIAB] OR triple[TIAB] OR tripled[TIAB] OR treble[TIAB] OR treble[TIAB]) AND (blind*[TIAB] OR mask*[TIAB])) OR ("4 arm"[tiab] OR "four arm"[tiab])OR clinical trial*[tiab]	1512638
#4	Search cohort studies[mesh:noexp] OR longitudinal studies[mesh:noexp] OR follow-up studies[mesh:noexp] OR prospective studies[mesh:noexp] OR retrospective studies[mesh:noexp] OR cohort[TIAB] OR longitudinal[TIAB] OR prospective[TIAB] OR retrospective[TIAB]	2406332
#5	Search CROSS-SECTIONAL STUDIES[MH] OR CASE-CONTROL STUDIES[MH] OR CROSS-SECTIONAL[TIAB] OR CASE-CONTROL[TIAB]	1397798
#6	Search #1 AND #2	26696
#7	Search #3 OR #4 OR #5	3991761
#8	Search #6 AND #7	7011
#9	Search #6 AND #7 Sort by: PublicationDate Filters: published in the last 10 years	4135
#10	Search #6 AND #7 Sort by: PublicationDate Filters: published in the last 10 years; English	**4024**
	**Embase Search**	

#1	((('kidney function'/exp OR 'renal function':ti,ab OR 'kidney function':ti,ab OR albuminuria:ti,ab OR proteinuria:ti,ab OR microalbuminuria:ti,ab OR 'glomerular filtration rate':ti,ab OR gfr:ti,ab OR kidney) AND function:ti,ab OR 'kidney dysfunction':ti,ab OR kidney:ti OR renal:ti OR 'urinary albumin-creatinine':ti,ab OR egfr:ti,ab OR 'renal insufficiency':ti,ab OR 'glomerular filtration rate':ti,ab OR renal) AND outcome:ti,ab OR 'renal outcomes':ti,ab OR 'proteinuria'/exp OR 'glomerulus filtration rate'/exp OR 'urinary albumin creatinine ratio'/exp) AND [2009-2019]/py AND [english]/lim	152942
#2	('diet quality' OR 'eating habit'/exp OR 'mediterranean diet'/exp OR dash:ab,ti OR 'dietary approaches to stop hypertension':ab,ti OR vegan*:ab,ti OR vegetarian*:ab,ti OR 'vegetarian diet'/exp OR 'vegetarian'/exp OR 'prudent diet':ab,ti OR 'western diet':ab,ti OR omniheart:ab,ti OR omni:ti OR 'plant based diet' OR ((dietary OR eating OR food OR diet) NEAR/2 (pattern? OR habit? OR profile? OR intake? OR recommendation? OR guideline?)) OR (('ethnic, racial and religious groups'/exp OR okinawa*) AND ('diet'/exp OR 'eating'/exp OR 'food intake'/exp))) AND [english]/lim AND [2009-2019]/py	72611
#3	#1 AND #2	947
#4	'case control study'/exp OR 'observational study'/exp OR 'cohort analysis'/exp OR 'clinical trial'/exp OR 'clinical trial':ti,ab OR cohort:ti,ab OR observational:ti,ab OR retrospective:ti,ab OR prospective:ti,ab OR longitudinal:ti,ab OR 'longitudinal study'/exp OR 'follow-up studies':ti,ab OR 'follow-up study':ti,ab OR 'case-control':ti,ab OR 'case control':ti,ab OR 'cross sectional':ti,ab OR 'randomization'/exp OR randomization:ti,ab OR randomized:ti,ab	4225057
**#5**	#3 AND #4	**440**

Ajjarapu et al. Dietary patterns and renal health outcomes in the general population: a review focusing on prospective studies. Online Supporting Information.

Supplemental Table 2. Characteristics of cross-sectional studies of dietary patterns and renal outcomes

First author, publication year, country	Population, sample size (sex)	Age, years	Outcome ascertainment	Diet-assessment method (no. of items)	Outcome (definition)	Dietary Pattern identified (method used)	Association measures with renal outcomes (RR, OR, HR, β, and 95% CI)	Covariates in fully adjusted model
Mazidi et al. (2018) [1], USA	NHANES, 21,649 (both)	Mean age of study sample (95% CI): 45.9 (45.2, 46.3)	-Serum and urine creatinine measured via Jaffe Method using samples collected during 2005-12 NHANES cycles.<br>-Urine albumin measured via solid phase florescent immunoassay using samples collected during 2005-12 NHANES cycles.<br>-eGFR calculated via CKD-EPI equation	24-hr diet recall. Dietary pattern calculated via diet measured during 2005-12 NHANES cycles.	1.Prevalent CKD (eGFR <60 ml/min/1.73m^2)	1. Saturated-MUFA (PCA)<br>2. Minerals and Vitamins (PCA)<br>3. Cholesterol-PUFA (PCA)	**Prevalent CKD (OR):**<br>1.Saturated-MUFA pattern<br>Q1: 1.00 (ref.)<br>Q2: 1.05 (0.82, 1.35)<br>Q3: 0.80 (0.64, 1.01)<br>Q4: 0.83 (0.66, 1.03)<br>2. Minerals and Vitamins pattern<br>Q1: 1.00 (ref.)<br>Q2: 0.65 (0.53, 0.80)<br>Q3: 0.66 (0.53, 0.81)<br>Q4: 0.50 (0.40, 0.62)<br>3. Cholesterol-PUFA pattern<br>Q1:1.00 (ref.)<br>Q2: 0.85 (0.72, 1.00)<br>Q3: 0.96 (0.79, 1.18)<br>Q4: 0.85 (0.67, 1.00)	Age, gender, BMI, race, hypertension, diabetes, triglycerides, high density lipoprotein
Shi et al. (2016) [2], China	CHNS, 8,429 (both)	Mean age of study sample: 51 (SD: 15)	-Serum creatinine measured via Jaffe kinetic method from samples collected in 2009<br>-eGFR calculated via MDRD equation	24-hr diet recall. Dietary pattern calculated from diet info measured in 2009	1.Prevalent CKD (eGFR <60 ml/min/1.73m^2)	1. Traditional southern (factor analysis)<br>2. Modern (factor analysis)	**Prevalent CKD (OR):**<br>Traditional Southern:<br>Q1:1.00 (ref.)<br>Q2: 2.43 (1.70, 3.47)<br>Q3: 4.92 (3.49, 6.93)<br>Q4: 4.56 (3.18, 6.56)<br>P for trend: <0.001<br>Modern:<br>Q1: 1.00 (ref.)<br>Q2: 0.74 (0.57, 0.97)<br>Q3: 0.53 (0.39, 0.72)<br>Q4: 0.50 (0.36, 0.71)<br>P for trend: 0.001	Age, gender, energy intake, education, income, urbanization level, smoking, alcohol drinking, physical activity, overweight/obesity, hypertension, and diabetes
Paterson et al. (2018) [3], Ireland	INES, 1033 (women)	Mean age of study sample: 76 (SD: 8)	-eGFR calculated via serum creatinine values using CKD-EPI equation from blood samples collected between 2007-09	FFQ (170). Dietary pattern calculated using FFQ administered between 2007-09.	1. Prevalent CKD (eGFR <60 ml/min/1.73m^2)	1. Healthy (PCA)<br>2. Unhealthy (PCA)	**Prevalent CKD (OR):**<br>Healthy<br>Q1: 1.00 (ref.)<br>Q2: 0.69 (0.43, 1.09)<br>Q3: 1.04 (0.64, 1.69)<br>Q4: 0.90 (0.56, 1.43)<br>Q5: 0.87 (0.54, 1.39)<br>P for trend: 0.97<br>Unhealthy<br>Q1: 1.00 (ref.)<br>Q2: 1.84 (1.17, 2.89)<br>Q3: 1.44 (0.92, 2.25)	Age, BMI, presence of diabetes, presence of hypertension, ever smoking, presence/history of ischemic heart disease, presence/history of cerebrovascular accident and ever alcohol

							Q4: 1.87 (1.19, 2.95)<br>Q5: 2.62 (1.65, 4.15)<br>P for trend: <0.001	
Nettleton et al., (2008) [4]. USA	MESA, 5042 (both)	Age range of study sample: 45-85	-Urine albumin was measured by a protein analyzer and urine creatinine was measured by rate reflectance spectrophotometry using a urine sample collected at baseline.	FFQ. (120)<br>Dietary pattern calculated using FFQ administered at baseline. FFQ measured diet during the past year.	1. Microalbuminuria (UACR: 25-249 mg/g)<br>2. UACR	1.Fats and processed meats (PCA)<br>2.Vegetables and fish (PCA)<br>3.Beans, tomatoes, and refined grains<br>4. Whole grains and fruit	**Microalbuminuria (OR:**<br>Fats and processed meats:<br>Q1: 1.00 (ref.)<br>Q2: 0.88 (0.63, 1.23)<br>Q3: 1.25 (0.88, 1.76)<br>Q4: 1.27 (0.87, 1.85)<br>Q5: 1.29 (0.81, 2.04)<br>P for trend: 0.19<br>Vegetables and fish:<br>Q1: 1.00 (ref.)<br>Q2: 1.45 (1.04, 2.02)<br>Q3: 1.16 (0.82, 1.65)<br>Q4: 1.30 (0.91, 1.86)<br>Q5: 1.12 (0.74, 1.72)<br>P for trend: 0.94<br>Beans, tomatoes, and refined grains:<br>Q1: 1.00 (ref.)<br>Q2: 1.70 (1.23, 2.36)<br>Q3: 1.18 (0.83, 1.67)<br>Q4: 1.53 (1.07, 2.19)<br>Q5: 1.24 (0.81, 1.91)<br>P for trend: 0.46<br>Whole grains and fruit:<br>Q1: 1.00 (ref.)<br>Q2: 0.81 (0.59, 1.11)<br>Q3: 0.56 (0.40, 0.80)<br>Q4: 0.54 (0.37, 0.77)<br>Q5: 0.65 (0.45, 0.95)<br>P for trend: 0.04<br><br>**UACR (β):**<br>Fats and processed meats:<br>0.019 (SD: 0.02)<br>Vegetables and fish:<br>0.008 (SD:0.02)<br>Beans, tomatoes, and refined grains:<br>0.025 (SD: 0.02)<br>Whole grains and fruit:<br>-0.036 (SD: 0.01)	Age, gender, energy intake, race/ethnicity, education, active leisure-time physical activity, inactive leisure-time physical activity, current smoking status, smoking duration, current supplement use, and study center.
Crews et al. (2014) [5], USA	NIA-HANDLS, 2085 (both)	Mean age of study sample: 48	-Serum creatinine measured via modified kinetic Jaffe method and isotope dilution mass spectrometry<br>-microalbumin measured via immunoturbimetric assay using blood samples collected between 2004-08.<br>-eGFR calculated via	24-hr diet recall. Dietary pattern calculated from two 24-hr diet recalls administered 7-10 days apart between 2004-08.	1. Prevalent CKD (eGFR <60 ml/min/1.73m^2)	1. DASH (diet score)	**CKD (OR):**<br>Poverty<br>T1: 3.20 (1.72, 5.96)<br>T2: 2.85 (1.23, 6.63)<br>T3: 1.00 (ref.)<br>P for trend: 0.001<br><br>Non-Poverty<br>T1: 0.91 (0.45, 1.85)<br>T2: 0.98 (0.40, 2.37)<br>T3: 1.00 (ref.)<br>P for trend: 0.801	Age, gender, and race

			CKD-EPI equation					
Lee et al. (2017) [6], Korea	KNHANES, 2408 (both)	Mean age of study sample: 72.4 (SD: 5.1)	-Serum creatinine measured via isotope dilution mass spectrometry and enzymatic colorimetric method using samples collected between 2011-12<br>-eGFR calculated via CKD-EPI equation<br>-Urine albumin measured via turbidimetric assay using sample collected between 2011-12	Qualitative FFQ and 24-hr diet recall. Dietary pattern calculated via diet info collected from FFQ and 24-hr recall between 2011-12	1.Prevalent CKD (eGFR <60 ml/min/1.73m^2 or UACR ≥30mg/g)	1. U.S. DASH (diet score)<br>2. Korean DASH (diet score)	**CKD (OR):**<br>DASH-US<br>Low score: 0.0 (ref.)<br>High score: 0.78 (0.65, 0.94)<br>DASH-Korea<br>Low score: 0.0 (ref.)<br>High score: 0.95 (0.91, 0.99)	Age, gender, BMI, diabetes, hypertension, hyperlipidemia, active smoking, physical activity, myocardial infarction, and stroke history
Gopinath et al. (2013) [7] ,Australia	Blue Mountain Eye Study, 2686 (both)	Age of study sample: ≥ 49	-Serum creatinine measured via isotope dilution mass spectrometry from samples collected between 1992-94.<br>-eGFR calculated via MDRD equation	FFQ (145). Diet score calculated via FFQ administered at baseline between 1992-94	1. Prevalent CKD (eGFR <60 ml/min/1.73m^2)	1.TDS (diet score)	**CKD: (OR)**<br>Q1:1.0 (ref.)<br>Q2:0.68 (0.48, 0.97)<br>Q3:0.51 (0.35, 0.74)<br>Q4:0.59 (0.41, 0.85)<br>P for trend: 0.005	BMI, receipt of pension, smoking, serum total cholesterol, serum triglycerides, hypertension, and history of diagnosed diabetes
Xu et al. (2015) [8] ,Sweden	USLAM and PIVUS, 1942 (both)	Age range of study sample: 71-72	-Serum creatinine measured via spectrophotometry using Jaffe reaction using samples collected between 1991-95 for USLAM And 2001-04 for PIVUS.<br>-Serum cystatin calculated via latex enhanced reagent using samples collected between 1991-95 for USLAM And 2001-2004 for PIVUS.<br>-eGFR calculated via serum creatinine and cystatin values CKD-EPI	7- day dietary records (~1500). Diet score calculated from diet records during a study visit between 1991-95 for USLAM and 2001-04 for PIVUS.	1. eGFR	1.ADII (diet score)	**eGFR (β):**<br>-0.018 (-0.027, -0.009); p-value: <0.001	Age, gender, BMI energy intake, smoking status, physical activity, hypertension, diabetes, use of lipid-lowering medication, and whether the participants were from the USLAM or PIVUS study cohorts

			cystatin and creatinine equation					
Chrysohoou et al.(2010) [9], Greece	ATICCA, 1975 (both)	Age of study sample: >18	-Serum creatinine measured via colorimetric method using samples collected between 2001-02. -Creatinine clearance rate calculated via Cockcroft-Gault Formula	FFQ (not reported). Dietary pattern calculated from FFQ administered at study visit between 2001-02.	1.Creatinine Clearance Rate	1.MDS (diet score)	**Creatinine Clearance Rate (β)** 0.003 (SE: ±0.001); p-value: 0.06	Not indicated
Lin et al. (2010), [10], Taiwan	Study participants were Buddhist nuns in Taichung City, Taiwan, 102 (female)	Mean age of study sample: 46.6	-Serum creatine and albumin measured via Olympus AU-2700 and the SYSMEX XE-2100 from samples collected between 2006-07 -eGFR calculated via serum creatinine values using MDRD equation	-Not reported. Diet information collected between 2006-07	1. eGFR	1. Duration of diet intake by vegetarian diet	**eGFR (β):** -0.07 (CI not reported); p-value: 0.67	Not indicated
Liu et al. (2019) [11], Taiwan	Individuals who received health paid exams at health checkup center in Taipei Chi Hospital, 55, 113 (both)	Age of study sample: ≥ 40	-Serum creatinine measured via Jaffe method using samples collected between 2005-16 -eGFR calculated via CKD-EPI equation -Urine protein measured via automated urine analyzer using samples collected between 2005-16	-Food questionnaire (not reported). Dietary pattern calculated via diet info collected during study visit between 2005-16	1.Prevalnet CKD (eGFR ≤60mL/min / 1.73 m^2 or proteinuria)	1. Vegan (only consumes plant-based foods) 2.Ovo-lacto (consumes eggs or dairy or both but no other animal products) 3.Omnivore (consumes both plant and animal-based foods)	**CKD (OR)** Omnivore: 1.0 (ref.) Vegan: 0.86 (0.75, 0.97) Ovo-lacto vegetarian: 0.82 (0.77, 0.88)	Age, gender diabetes, hypertension, abdominal obesity, systolic BP, low HDL, and high TG

Abbreviation of research studies: HR=Hazard ratio; RR=Relative risk ratio; OR=Odd's ratio; β=beta correlation coefficient; SD=standard deviation; SE= standard error; FFQ=Food frequency questionnaire; UACR=urinary albumin-to-creatinine ratio; eGFR=estimated glomerular filtration rate; BMI= body mass index; PCA=Principle component factor analysis; T= Tertial; Q= Quartile or quintile based on designation; NHANES= US National Health and Nutrition Examination

Surveys; CHNS=China Health and Nutrition Survey; INES= Irish Nun Eye Study; CKD-EPI=Chronic Kidney Disease Epidemiology Collaboration; NIA-HANDLS=National Institute on Aging, Healthy Aging in Neighborhoods of Diversity across the Life Span; KNHANES=Korean National Health and Nutrition Examination Survey; ULSAM=Uppsala Longitudinal Study of Adult Men; PIVUS=Prospective Investigation of Vasculature in Uppsala Seniors; MESA=Multi-ethnic Study of Atherosclerosis; DASH=Dietary Approaches to Stop Hypertension; TDS=Total Diet Score; MDS=Mediterranean Diet Score; ADII=Adapted Dietary Inflammatory Index; MDRD= Modification in Diet and Renal Disease.

Supplemental Table 3. Description of *a priori* dietary patterns

Dietary Pattern: Study (First Author, Year of Publication)	Components of diet score	Diet score calculation
Dietary Approaches to Stop Hypertension:<br>Smyth et al., 2016<br>Asghari et al., 2017<br>Lin et al., 2011<br>Taylor et al., 2009<br>Ferraro et al., 2017<br>Rebholz et al., 2016	High intake of:<br>1. Vegetables<br>2. Fruit<br>3. Whole grains<br>4. Low-fat dairy products<br>5. Nuts & legumes<br>Low intake of:<br>6. Sugar sweetened beverages<br>7. Red/processed meat<br>8. Sodium (mg/day)	Component score range: 1-5 based on levels of intake.<br>Total Score: sum of component scores with range of 8-40.<br>Higher scores indicate greater adherence.
Dietary Approaches to Stop Hypertension:<br>Chang et al., 2013	High intake of:<br>1. Vegetables<br>2. Fruit<br>3. Whole grains<br>4. Low-fat dairy products<br>5. Nuts & legumes<br>Low intake of:<br>6. Sugar sweetened beverages<br>7. Red/processed meat<br>8. Sodium (mg/day)	Component score range: 1-4 based on quartile of intake.<br>Total score: sum of component scores with range of 8-32.<br>Higher scores indicate greater adherence.
Dietary Approaches to Stop Hypertension:<br>Liu et al, 2017<br>Crews et al., 2017	1. Total fat<br>2. Saturated fat<br>3. Protein<br>4. Fiber<br>5. Cholesterol<br>6. Calcium<br>7. Magnesium<br>8. Potassium<br>9. Sodium	Component score range: 0-1 based on meeting a target intake.<br>Total score: sum of component scores with range of 0-9.<br>Higher scores indicate greater adherence.
Dietary Approaches to Stop Hypertension-US and Dietary Approaches to Stop Hypertension-KQ:<br>Lee et al., 2017	1. Protein<br>2. Fiber<br>3. Calcium<br>4. Potassium<br>5. Total Fat	Component score range: 0-1 based on meeting a target intake for DASH-US and 1-4 based on quartile of intake for DASH-KQ.

	6. Sodium	Total score: sum of component scores with range of 0-6 for DASH-US and 6-24 for DASH-KQ.<br>Higher scores indicate greater adherence.
Mediterranean Diet:<br>Khatri et al., 2014<br>Smyth et al., 2016<br>Leone et al., 2017	High intake of:<br>1. Legumes<br>2. Vegetables<br>3. Fruit<br>4. Cereals<br>5. Legumes<br>6. Fish<br>Moderate to high intake:<br>7. MUFA to SFA<br>Moderate intake:<br>8. Alcohol<br>Low intake of:<br>9. Dairy and Meat	Component score range: 0-1 based on levels of intake.<br>Total score: sum of component scores with range of 0-9.<br>Higher scores indicate greater adherence.
Mediterranean Diet:<br>Asghari et al., 2017	High intake of:<br>1. Legumes<br>2. Vegetables<br>3. Nuts and fruits<br>4. Cereals<br>5. MUFA to SFA<br>Moderate to high intake of:<br>6. Fish<br>Low to moderate intake of:<br>7. Dairy products<br>Low intake of:<br>8. Meat and poultry	Component score range: 0-1 based on levels of intake<br>Total Score: sum of component scores with range of 0-8.<br>Higher scores indicate greater adherence.
Mediterranean Diet:<br>Chrysohoou et al., 2010	High intake of:<br>1. Non-refined cereals<br>2. Fruits<br>3. Vegetables<br>4. Legumes<br>5. Olive oil<br>6. Fish<br>7. Potatoes<br>Low intake of:<br>8. Red meat and products<br>9. Poultry<br>10. Full fat dairy products<br>11. Alcohol	Component score range: 0-5 based on levels of intake.<br>Total score: sum of component scores with range of 0-55.<br>Higher scores indicate greater adherence.

Alternative Healthy Eating Index-2010:<br>Smyth et al., 2016	High intake of:<br>1. Vegetables<br>2. Fruit<br>3. Whole Grains<br>4. Nuts, legumes and vegetable protein<br>5. Long-chain (n-3) fats (EPA+DHA)<br>6. PUFA<br>Moderate intake of:<br>7. Alcohol<br>Low intake of:<br>8. Sugar-sweetened beverages<br>9. Red meat and processed meats<br>10. *Trans* fat<br>11. Sodium	Component score range: 0-10 based on levels of intake.<br>Total score: sum of component scores with range of 0-110.<br>Higher scores indicate greater adherence.
Healthy Eating Index-2010:<br>Smyth et al., 2016	High intake of:<br>1. Total vegetables<br>2. Greens & beans<br>3. Total fruit<br>4. Whole fruit<br>5. Whole grains<br>6. Dairy<br>7. Total protein<br>8. Seafood and plant proteins<br>9. Fatty acids<br>Low intake of:<br>10. Refined grains<br>11. Sodium<br>12. Empty calories (calories from solid fats, alcohol, and added sugars)	Component score range: 1-10 (whole grains, dairy, fatty acids, refined grains, and sodium), 0-20 (empty calories), 0-5 (rest of components) based on levels of intake.<br>Total score: sum of components scores with range of 0-100.<br>Higher scores indicate greater adherence.
Recommended Food Score:<br>Smyth et al., 2016	High intake of:<br>1-9.Vegetable items<br>10-15. Fruit items<br>16-20.Whole grains<br>21-22. Poultry Items<br>23. Fish item	Component score range: 0-1 based on levels of intake.<br>Total score: sum of component scores with range of 0-23.<br>Higher score indicate greater adherence.

Dietary Guidelines Adherence Index:<br>Foster et al., 2015	1) Dark green vegetables<br>2) Orange vegetables<br>3) Legumes<br>4) Starchy vegetables<br>5) Other vegetables<br>6) Fruits<br>7) Variety of fruits and vegetables<br>8) Meats and legumes<br>9) Milk and milk products<br>10) Grains<br>11) Discretionary energy (added sugar intake)<br>12) Whole grains<br>13) Fiber<br>14) Low-fat choices<br>15) Total fat<br>16) Saturated fat<br>17) *Trans* fat<br>18) Cholesterol<br>19) Alcohol<br>20) Sodium	Component score range: 0-1 based on level of intake.<br>Total score: sum of component scores with range of 0-20.<br>Higher scores indicate greater adherence.
American Heart Association's Healthy Diet Score:<br>Rebholz et al., 2015	High intake of:<br>1. Fruits and Vegetables<br>2. Fish<br>3. Fiber-rich whole grains<br>Low intake of:<br>4. Sodium<br>5. Sugar-sweetened beverages	Component score range: 0-1.<br>Total score: sum of component scores with range of 0-5.<br>Higher scores indicate greater adherence.
Total Diet Score:<br>Gopinath et al., 2013	1. Eat plenty of vegetables, legumes and fruit<br>2. Eat plenty of cereals, preferably wholegrain/meal<br>3. Include lean meats, fish, poultry and/or alternatives<br>4. Include milk, yoghurts, cheese, and/or alternatives<br>5. Limit saturated fat and moderate total fat intake	Component score range: 0-2 based on meeting an intake recommendation.<br>Total score: sum of component scores with range of 0-20.<br>Higher scores indicate greater adherence.

	6. Choose foods low in salt<br>7. Limit alcohol intake if you choose to drink<br>8. Consume only moderate amounts of sugars and foods with added sugars<br>9. Extra foods, not essential to provide nutrients and may be high in salt, fat or sugar<br>10. Prevent weight gain: be physically active and eat according to energy needs	
Adapted Dietary Inflammatory Index:<br>Xu et al., 2015	1. Protein<br>2. Total fat<br>3. Saturated fatty acid<br>4. MUFAs<br>5. n-3 PUAS<br>6. Cholesterol<br>7. Carbohydrate<br>8. Fiber<br>9. Ethanol<br>10. Caffeine<br>11. Vitamin A<br>12. Beta-carotene<br>13. Thiamin<br>14. Riboflavin<br>15. Niacin<br>16. Vitamin B6<br>17. Folate<br>18. Vitamin B12<br>19. Vitamin C<br>20. Vitamin D<br>21. Vitamin E<br>22. Iron<br>23. Magnesium<br>24. Selenium<br>25. Zinc<br>26. Tea	Total score: product of the dietary inflammatory weights of 26 individual components.
Vegetarian:<br>Turney et al., 2014	Does not eat meat or fish	Categorized as vegetarian if didn't eat meat or fish as indicated on questionnaire.

Omnivore, Vegan, Ovo-lacto Vegetarian:<br>Liu et al., 2019	Omnivore: consumes both plant and animal-based foods<br>Vegan: only consumes plant-based foods<br>Ovo-lacto vegetarian: consumes eggs or dairy products or both but no other animal products.	Dietary patterns were determined by responses to validated food questionnaire.

Ajjarapu et al. Dietary patterns and renal health outcomes in the general population: a review focusing on prospective studies. Online Supporting Information.

Supplemental Table 4. Description of *a posteriori* dietary patterns

Dietary pattern: Study (First Author, Year of Publication)	Dietary pattern description	How dietary pattern was derived in study
Prudent Pattern:<br>Lin et al., 2011	High intake of fruits, vegetables, legumes, fish, poultry, and whole grains.	Principle component procedure identifies diet patterns based on correlations between 38 food groups classified from FFQ.
Western Pattern:<br>Lin et al., 2011	High intake of red and processed meats, saturated fats, and sweets.	Principle component procedure identifies diet patterns based on correlations between 38 food groups classified from FFQ.
Lacto-vegetarian:<br>Asghari et al., 2018	High intake of fresh fruit, dried fruit and fruit juice, dark-yellow, and leafy vegetables, tomato, date, low-fat dairy, and olive oil.	Principal component factor analysis identified dietary patterns from response from the food frequency questionnaire .
Traditional Iranian:<br>Asghari et al., 2018	High intake of legumes processed and red meat, potato, egg, refined grain, sugar, French fries, and tea.	Principal component factor analysis identified dietary patterns from response from the food frequency questionnaire .
High fat, high sugar:<br>Asghari et al., 2018	High intake of mayonnaise, coffee, sweet and salty snack, soda, high-fat dairy, pizza, butter, salt, solid oil, poultry, and corn and peas.	Principal component factor analysis identified dietary patterns from response from the food frequency questionnaire.
Saturated-MUFA:<br>Mazidi et al., 2018	Defined by saturated fatty acids, mono-unsaturated fatty acids, total fat, and carbohydrate	Principle component analysis was used to generate dietary patterns from responses form the food frequency questionnaire.
Minerals and Vitamins:<br>Mazidi et al., 2018	Defined by vitamins, minerals, and dietary fiber	Factor analysis with orthogonal transformation was used to derive nutrient patterns based on nutrients and bioactive compounds from responses from dietary recalls.
Cholesterol-PUFA:<br>Mazidi et al., 2018	Defined by cholesterol, polyunsaturated fatty acids, and protein	Factor analysis with orthogonal transformation was used to derive nutrient

		patterns based on nutrients and bioactive compounds from responses from dietary recalls.
Traditional Southern:<br>Shi et al., 2016	Defined by rice, pork, and vegetable	Factor analysis was used to generate dietary patterns from food frequency questionnaire responses.
Modern:<br>Shi et al., 2016	Defined by fruit, soy milk, eggs, milk, deep fried products, fast food and cakes	Factor analysis was used to generate dietary patterns from food frequency questionnaire responses.
Healthy:<br>Paterson et al., 2018	Defined by lutein/zeaxanthin-rich vegetables, green leafy vegetables, alliums, vegetables, fruit, tomatoes, legumes, nuts, oily fish, low fat dairy products, pizza, dressings/sauces/condiments, wholegrain breakfast cereal and red meat.	Principle component analysis was used to generate dietary patterns from responses form the food frequency questionnaire.
Unhealthy:<br>Paterson et al., 2018	Defined by crisps, chips, alcohol, high fat dairy products, soups, desserts, sugars and sweets, wholegrains, dressings/sauces/condiments, processed meat, potatoes, eggs, refined grains, refined breakfast cereal, chocolate vegetables, red meat, white fish and shell fish.	Principle component analysis was used to generate dietary patterns from responses of food frequency questionnaire.
Fats and processed meats:<br>Nettleton et al., 2008	Described as added fats, processed meat, fried potatoes, and desserts.	Principle component analysis was used to generate dietary patterns from responses of food frequency questionnaire.
Vegetables and fish:<br>Nettleton et al., 2008	Vegetables, fish, soups, and Chinese dishes.	Principle component analysis was used to generate dietary patterns from responses of food frequency questionnaire.
Beans, tomatoes, and refined grains:<br>Nettleton et al., 2008	Beans, tomatoes, refined grains, high-fat dairy foods, red meat, and poultry.	Principle component analysis was used to generate dietary patterns from responses of food frequency questionnaire.

Whole grains and fruit: Nettleton et al., 2008	Whole grains, fruit, nuts and seeds, green leafy vegetables, and low-fat dairy foods.	Principle component analysis was used to generate dietary patterns from responses of food frequency questionnaire.

REFERENCES

1. Mazidi, M.; Gao, H.-k.; Kengne, A.P. Food Patterns are Associated with Likelihood of CKD in US Adults. *Scientific Reports* **2018**, *8*, 10696, doi:10.1038/s41598-018-27365-6.
2. Shi, Z.; Taylor, A.W.; Riley, M.; Byles, J.; Liu, J.; Noakes, M. Association between dietary patterns, cadmium intake and chronic kidney disease among adults. *Clin Nutr* **2018**, *37*, 276-284, doi:10.1016/j.clnu.2016.12.025.
3. Paterson, E.N.; Neville, C.E.; Silvestri, G.; Montgomery, S.; Moore, E.; Silvestri, V.; Cardwell, C.R.; MacGillivray, T.J.; Maxwell, A.P.; Woodside, J.V., et al. Dietary patterns and chronic kidney disease: a cross-sectional association in the Irish Nun Eye Study. *Scientific reports* **2018**, *8*, 6654, doi:10.1038/s41598-018-25067-7.
4. Nettleton, J.A.; Steffen, L.M.; Palmas, W.; Burke, G.L.; Jacobs, D.R., Jr. Associations between microalbuminuria and animal foods, plant foods, and dietary patterns in the Multiethnic Study of Atherosclerosis. *The American journal of clinical nutrition* **2008**, *87*, 1825-1836, doi:10.1093/ajcn/87.6.1825.
5. Crews, D.C.; Kuczmarski, M.F.; Miller, E.R., 3rd; Zonderman, A.B.; Evans, M.K.; Powe, N.R. Dietary habits, poverty, and chronic kidney disease in an urban population. *Journal of renal nutrition : the official journal of the Council on Renal Nutrition of the National Kidney Foundation* **2015**, *25*, 103-110, doi:10.1053/j.jrn.2014.07.008.
6. Lee, H.S.; Lee, K.B.; Hyun, Y.Y.; Chang, Y.; Ryu, S.; Choi, Y. DASH dietary pattern and chronic kidney disease in elderly Korean adults. *European journal of clinical nutrition* **2017**, *71*, 755-761, doi:10.1038/ejcn.2016.240.
7. Gopinath, B.; Harris, D.C.; Flood, V.M.; Burlutsky, G.; Mitchell, P. A better diet quality is associated with a reduced likelihood of CKD in older adults. *Nutrition, metabolism, and cardiovascular diseases : NMCD* **2013**, *23*, 937-943, doi:10.1016/j.numecd.2012.07.003.
8. Xu, H.; Sjogren, P.; Arnlov, J.; Banerjee, T.; Cederholm, T.; Riserus, U.; Lindholm, B.; Lind, L.; Carrero, J.J. A proinflammatory diet is associated with systemic inflammation and reduced kidney function in elderly adults. *The Journal of nutrition* **2015**, *145*, 729-735, doi:10.3945/jn.114.205187.
9. Chrysohoou, C.; Panagiotakos, D.B.; Pitsavos, C.; Skoumas, J.; Toutouza, M.; Papaioannou, I.; Stefanadis, C. Renal function, cardiovascular disease risk factors' prevalence and 5-year disease incidence; the role of diet, exercise, lipids and inflammation markers: the ATTICA study. *QJM : monthly journal of the Association of Physicians* **2010**, *103*, 413-422, doi:10.1093/qjmed/hcq045.
10. Lin, C.K.; Lin, D.J.; Yen, C.H.; Chen, S.C.; Chen, C.C.; Wang, T.Y.; Chou, M.C.; Chang, H.R.; Lee, M.C. Comparison of renal function and other health outcomes in vegetarians versus omnivores in Taiwan. *Journal of health, population, and nutrition* **2010**, *28*, 470-475.
11. Liu, H.W.; Tsai, W.H.; Liu, J.S.; Kuo, K.L. Association of Vegetarian Diet with Chronic Kidney Disease. *Nutrients* **2019**, *11*, doi:10.3390/nu11020279.
